# Analysis of Positional Physical Demands in Tier 2 Rugby Union: A Multivariate Approach over Speed Ranges

**DOI:** 10.3390/sports13080260

**Published:** 2025-08-08

**Authors:** Angel Lino-Samaniego, Adrián Martín-Castellanos, Ignacio Refoyo, Mar Álvarez-Portillo, Matthew Blair, Diego Muriarte Solana

**Affiliations:** 1 Facultad de Ciencias de la Actividad Física y del Deporte (INEF-Departamento de Deportes), Universidad Politécnica de Madrid, C/Martín Fierro, 7, 28040 Madrid, Spain; adrimaca@uax.es (A.M.-C.); ignacio.refoyo@upm.es (I.R.); diego.muriarte@upm.es (D.M.S.); 2 Department of Physical Activity and Sports Science, Alfonso X El Sabio University (UAX), 28691 Madrid, Spain; maralvarezportillo@gmail.com; 3 Spanish Rugby Federation, 28008 Madrid, Spain; 4 Institute of Sport, Exercise and Health, Otago Polytechnic, Dunedin 28040, New Zealand; mat.blair@op.ac.nz

**Keywords:** cluster analysis, global positioning system, injury prevention, performance, principal component analysis, team sport, workload monitoring

## Abstract

Rugby union involves intermittent high- and low-intensity activities, making it essential for strength and conditioning practitioners to understand specific physical demands. While GPS technology has enhanced this understanding, limited research focuses on Tier 2 national teams. This study aimed to describe the speed-related physical demands of a Tier 2 national rugby union team. This retrospective observational study analyzed 230 GPS files from 55 professional male players of an international Tier 2 national rugby union team, collected across 17 international matches. Speed-related performance variables were analyzed. Players who played ≥55 min were included. A Kruskal–Wallis test with post hoc comparisons was used to examine positional differences. Principal Component Analysis (PCA) identified four main components explaining 84.65% of the variance, while a two-step cluster analysis grouped players into Low-, Mid-, and High-Demand profiles based on these components. Backs showed greater high-intensity running demands compared to forwards. This study’s results provide novel insights into the physical demands of Tier 2 international rugby union, highlighting differences among player positions and clustering players based on their specific speed demands. These findings can help strength and conditioning practitioners design position-specific training loads, implement tailored recovery strategies, and reduce injury risk in Tier 2 international rugby union.

## 1. Introduction

Rugby union is a field-based contact team sport, characterized by repeated periods of high- and low-intensity activities [1,2]. The intermittent nature of the sport is defined by brief bouts of high-intensity exercise such as running, sprinting, tackling, rucking, etc., interleaved with low- or moderate-intensity activities such as standing, walking, or jogging [3].

Rugby union strength and conditioning coaches and other sports professionals have a great interest in understanding the specific physical match demands of rugby union for the design of effective training programs that might enhance players’ performance [4]. GPS (Global Positioning System) technology has enabled sports scientists to gather this specific physical knowledge, which can help quantify training and match demands [5]. This kind of technology provides information such as the distance players travel during a training session or match and the distance at various speed thresholds [5,6].

Previous research has explored the physical demands of rugby union through various methodological approaches and across different playing levels. Studies have focused on both senior and youth players [7], highlighting the variability in demands across age groups. Specific aspects such as repeated high-intensity efforts (RHIE) [8], ball-in-play periods (BiP) [2], and worst-case scenarios (WCS) [9] have been used to analyze peak performance loads. In addition, alternative training formats like small-sided games (SSG) have also been examined for their potential to replicate a match [10,11,12].

Principal Component Analysis (PCA) is currently used to reduce the dimensionality of large datasets by transforming a large set of variables into a smaller one that still contains most of the information in the large set. In rugby union, PCAs have been used to assess different anthropometric parameters of body regions [13] or separate principal components (PCs) of training load and intensity [14]. They have also been used to identify PCs from internal and external loads combined with skills [15].

Cluster analyses are commonly carried out to identify and group similar data points from a large dataset. In rugby union, these analyses have been performed to classify players based on different conditioning-specific performance tasks [16] or to compare the match characteristics by age categories and playing standard [17]. The identification of biomechanical variables related to successful place kicking has also been studied with cluster analysis by creating a 13-segment biomechanical model of placekickers with 6 degrees of freedom at each joint and a one-segment ball model with 6 degrees of freedom [18].

Although the physical demands of the best national and international competitions of professional rugby union are well studied and established, there has not been any investigation studying lower levels of rugby union. Research is yet to examine the physical running demands of Tier 2 (national teams ranked between 12 and 24 by World Rugby).

In addition, the physical and physiological demands vary greatly depending on player position, with forwards and backs experiencing distinct intensity profiles, movement patterns, and workloads [4,19]. As such, individualized training based on positional requirements is crucial to optimize performance, guide recovery strategies, and reduce injury risk. Understanding these position-specific demands in Tier 2 competition is essential for practitioners working at this level.

Therefore, the purpose of this research was to describe the physical demands of a Tier 2 national team by studying the speed performance parameters.

## 2. Materials and Methods

### 2.1. Article Type

In the present research, the international rugby union performance of the Spanish Men’s Senior National team in the European Rugby Championship (REC) facing Tier 2 and 3 teams was analyzed (i) to recognize differences of specific player positions in speed performance parameters, (ii) to understand the principal components of speed performance parameters, and (iii) to create a model of position profiles through the principal components (PCs) of the speed performance parameters.

### 2.2. Participants

Fifty-five (n = 55) male rugby union Tier 2 level players (185 ± 8 cm; 96.75 ± 14.01 kg) from the Spanish Men’s Senior National team participated in the study (Table 1).

A professional status was required for all players, and a minimum of 55 min of playing time was applied as an inclusion criterion [8,20]. Players were excluded if they were not professional, were under any pharmacological medical treatment, or failed to meet the 55 min playing time threshold. None of the participants reported any musculoskeletal injury or disease during the data collection period. Players were classified according to specific playing positions as Prop (loose-head and tight-head; n = 6), Hooker (n = 5), Second-row (n = 7), Back-row (blind side, open side and No 8; n = 9) comprising the forwards and Scrum-half (n = 5), Fly-half (n = 4), Centre (n = 6), and Back-three (wings and fullback; n = 13) comprising the backs [8]. These authors also suggested that players positions such as No 8 and flankers were grouped in the Back-row position, as well as wings and fullbacks in the Back-three position.

### 2.3. Procedures

Seventeen official matches from 2019 (n = 5), 2020 (n = 2), 2021 (n = 5), and 2022 (n = 5) were included in the study, resulting in a total of N = 426 GPS files. However, just n = 230 GPS files were included in the study because of the 55 min playing time inclusion criterion. All matches took place between February and March (except 2021 matches), between 12 p.m. and 6 p.m. Of the seventeen matches studied, there were 12 wins and 5 losses. In accordance with the Declaration of Helsinki, all data were obtained as part of routine measurements of the competitive stage, considered a condition of the duties of the professional athlete [21]. Nevertheless, ethical approval for research involving humans was obtained from the Ethics Committee of the Universidad Alfonso X El Sabio (2024_12/311; 20 January 2025). Additionally, all participants provided written informed consent prior to data inclusion.

All data were collected using Catapult Vector S7 (Catapult Innovation, Melbourne, Australia) sampling at 10 Hz and integrating a tri-axial accelerometer, a gyroscope, and a magnetometer, all sampling at 100 Hz. Catapult technology has been used to study speed thresholds in rugby union [22]. Nowadays, the accuracy and reliability of the GPS and IMU technologies have improved the sampling rates from 1 Hz to 10–15 Hz [21]. The reliability inter-unit appears to be linked to the higher sampling frequency, demonstrating that units sampling at 10 Hz are sufficiently accurate to quantify team sports metrics as velocity running phases, accelerations, or decelerations [9]; 10 Hz GPS units have been reported two to three times more accurate for instantaneous velocity during tasks completed at a range of velocities compared to a criterion measure [23].

The GPS devices were placed in a specific Catapult vest, which ensured the placement of the device between the player’s shoulder blades, in the upper region of the thoracic spine. All players wore the vest and GPS unit in training to facilitate the familiarization [19]. The GPS devices were turned on 75 min before the start of the match and left outside in the open sky to ensure a completely stable connection between the GPS devices and satellites, according to the manufacturer’s guidelines [9,24]. Data obtained from the GPS devices were treated with Openfield Cloud 4.7 software (Catapult Innovations, Melbourne, Australia) to determine the duration of the matches. The spreadsheet software Excel (Microsoft Office 2022) was used to create the base data.

### 2.4. Study Variables

Variables were not split into game sections, e.g., First vs. Second Half, as traditionally reported. Instead, the GPS performance metrics from the entire match were captured (Table 2). These variables were divided into general speed parameters (i) as Played Time (minutes), Total Distance (meters), Meters per Minute (meters/minute), PlayerLoad (arbitrary units, AU), or PlayerLoad per Minute (AU/meters) and low-intensity speed parameters (ii) or high-speed parameters (iii), both studying Distance and Effort Bouts in different speed bands. In addition, Maximum Velocity (km/h) and Sprint Distance (%) were included in high-speed parameters. Speed thresholds were defined as Band 1 (0–7 km/h), Band 2 (7–10.8 km/h), Band 3 (10.8–15 km/h), Band 4 (15–18 km/h), Band 5 (18–21.6 km/h), Band 6 (21.6–25.2 km/h), Band 7 (25.2–30 km/h), and Band 8 (30–39.6 km/h), similar to other studies [4,25]. The selection of the velocity bands was informed by current practices within the Spanish Rugby Federation and aligns with recommendations from World Rugby, allowing for detailed differentiation of performance intensity levels and facilitating applied analysis in the elite rugby union context.

### 2.5. Statistical Analysis

The normality of the data was assessed using the Shapiro–Wilk test and the homogeneity of variance using the Levene test. Outlier values corresponding to ±1.5 times the interquartile distance were removed from the study. Considering the non-compliance with these suggestions, a non-parametric Kruskal–Wallis test was performed to determine the effect of players’ specific positions on the speed performance variables. Post hoc analysis of Dwass–Steel–Critchlow–Fligner was applied to identify significant differences between the groups for each parameter studied. The interpretations of η^2^ were based on the following values: small, η^2^ < 0.06; medium, 0.06 ≤ η^2^ < 0.14; and large, η^2^ ≥ 0.14 [26].

Principal Component Analysis (PCA) was performed to extract the principal components (PCs) of the speed performance parameters using orthogonal Varimax rotation and to reduce the dimensions of data that have a large number of interrelated continuous quantitative variables while preserving the maximal [27,28]. Factors that did not reach one in the eigenvalue were not included in the study. The *Kaiser*–*Meyer*–*Olkin* test was conducted to measure sampling adequacy, resulting in a value of 0.601, indicating acceptable sampling adequacy [29]. Furthermore, Bartlett’s sphericity test was checked (χ^2^ = 10380.75; df = 231; *p* < 0.001).

A two-stage cluster was performed using twenty-two entries corresponding to the continuous quantitative PCs obtained in the PCA , aiming to reduce multicollinearity and dimensionality of the analysis. The objective of this technique was to group player positions with similar performance profiles, maximizing intragroup homogeneity and intergroup diversity over the speed performance parameters. This technique helped reveal patterns and structures within a dataset that can provide insight into the underlying relationships. The number of clusters was automatically determined based on the Bayesian Information Criterion (BIC), as implemented by SPSS using Schwarz’s method. The quality of the cluster solution was measured with the silhouette coefficient, which resulted in a regular 0.4 [30]. The importance of each variable in the clustering solution was estimated automatically by the algorithm and expressed as a normalized value between 0 and 1, where higher values indicate greater relevance for cluster separation.

Correspondence analyses were conducted to examine the connection between specific players’ positions and the different groups obtained after the cluster analysis. The use of this method provides a graphical portrayal, particularly useful in revealing the nature of the relationship between the studied objects [26]. Data were analyzed with IBM SPSS software (version 26.0, Armonk, NY, USA) and Jamovi (version 2.3.28, The Jamovi Project, 2022). The level of statistical significance was established at *p* < 0.05 for all the statistical tests.

## 3. Results

### 3.1. Positional Differences

The Kruskal–Wallis test showed significant differences in all the studied variables. The post hoc analysis of Dwass–Steel–Critchlow–Fligner was performed to identify statistical differences between the specific groups for each parameter studied (Table 3). The player’s specific positions evidenced statistical differences between forwards and backs.

The general speed parameters revealed significant differences (Played Time (F_7_ = 54.0; *p* < 0.001; η^2^ = 0.236), Total Distance (F_7_ = 72.9; *p* < 0.001; η^2^ = 0.318), Meters Per Minute (F_7_ = 69.8; *p* < 0.001; η^2^ = 0.305), Total PlayerLoad (F_7_ = 45.5; *p* < 0.001; η^2^ = 0.200), PlayerLoad Per Minute (F_7_ = 31.5; *p* < 0.001; η^2^ = 0.139)). Within the forward group, Props showed statistically less PlayerLoad per Minute than Hookers, less Played Time, Total Distance, and Total Playerload than Second-row players, and had less Played Time, Total Distance, Meters per Minute, and Total PlayerLoad than Back-row players. Moreover, Hookers presented statistically lower values in Played Time than Second-row players, but more Meters per Minute and PlayerLoad per Minute. Hookers had statistically less Played Time and Total Distance than Back-row players. In addition, Second-row players revealed statistically lower values in Total Distance, Meters per Minute, PlayerLoad, and PlayerLoad per Minute than Back-row players.

On the other hand, in the backs group, Scrum-halves did not present statistical differences with Fly-halves except for lower values in Meters per Minute. Scrum-halves did not show any significant differences either with Centres or Back-three players. Fly-halves ran statistically more than Centres in Meters per Minute. No other differences were found between Fly-halves, Centres, and Back-three players.

Kruskal–Wallis analysis revealed significant differences for low-intensity speed parameters (Table 3) (Velocity Band 1 Distance (F_7_ = 64.2; *p* < 0.001; η^2^ = 0.280), Velocity Band 2 Distance (F_7_ = 26.3; *p* < 0.001; η^2^ = 0.115), Velocity Band 2 Effort Count (F_7_ = 42.7; *p* < 0.001; η^2^ = 0.187), Velocity Band 3 Distance (F_7_ = 30.3; *p* < 0.001; η^2^ = 0.132), Velocity Band 3 Effort Count (F_7_ = 58.1; *p* < 0.001; η^2^ = 0.254), Velocity Band 4 Distance (F_7_ = 100.3; *p* < 0.001; η^2^ = 0.438), Velocity Band 4 Effort Count (F_7_ = 93.6; *p* < 0.001; η^2^ = 0.409)). For the forward positions, the post hoc analysis showed that Props performed statistically fewer meters than Hookers in Velocity Band 4 Distance and Velocity Band 3 Effort Count, had lower values in Velocity Band 4 Distance and in Velocity Band 2-3-4 Effort Count than Second-row players, and showed lower values in Velocity Band 1-2-3-4 Distance and Velocity Band 2-3-4 Effort Count than the Back-row players. Hookers did not show statistical differences for any low-intensity speed parameters with Second-row players, but revealed lower values in Velocity Band 1 and 4 Distance and Velocity Band 2 Effort Count than the Back-row players. Second-row players only performed statistically less than Back-row players in Velocity Band 1 Distance and in Velocity Band 3 Effort Count.

Within the backs group, Scrum-halves did not show statistical differences for low-intensity speed parameters with Fly-halves but revealed lower values than Centres and Back-three players in Velocity Band 1 Distance. Fly-halves did not present statistical differences either with Centres or the Back-three players, and Centres did not show any significant difference in low-intensity speed parameters when compared to Back-three players.

As presented in Table 3, the high-intensity speed parameters showed significant differences as well (Velocity Band 5 Distance (F_7_ = 107.2; *p* < 0.001; η^2^ = 0.468), Velocity Band 5 Effort Count (F_7_ = 100.8; *p* < 0.001; η^2^ = 0.440), Velocity Band 6 Distance (F_7_ = 125.4; *p* < 0.001; η^2^ = 0.548), Velocity Band 6 Effort Count (F_7_ = 110.0; *p* < 0.001; η^2^ = 0.480), Velocity Band _7_ Distance (F_7_ = 133.5; *p* < 0.001; η2 = 0.583), Velocity Band 7 Effort Count (F_7_ = 128.3; *p* < 0.001; η^2^ = 0.560), Velocity Band 8 Distance (F_7_ = 46.9; *p* < 0.001; η^2^ = 0.231), Velocity Band 8 Effort Count (F_7_ = 47.9; *p* < 0.001; η^2^ = 0.221), Maximum Speed (F_7_ = 157; *p* < 0.001; η^2^ = 0.686), Sprint Distance (F_7_ = 119.6; *p* < 0.001; η^2^ = 0.522)). In the forward group, post hoc analysis revealed no statistical differences between Props and Hookers in any high-intensity speed parameter but showed lower values for Props compared with Second-row players in Velocity Band 5-6 Distance and in Velocity 5 Effort Count and lower values than the Back-row players in Velocity Band 5-6 Distance, Velocity Band 5-6 Effort Count, Maximum Velocity, and Sprint Distance. Also, Hookers performed significantly less than Second-row players in Maximum Velocity and had lower values than the Back-row players in Velocity Band 5-6 Distance and Velocity Band 5-6 Effort Count. Second-row players evidenced significantly lower values in Velocity Band 5 Distance, Velocity Band 5 Effort Count, and Maximum Velocity compared to the Back-row players.

For the backs, the post hoc analysis showed statistically lower values in Maximum Velocity for Scrum-halves compared with Fly-halves and lower values than the Back-three players in Velocity Band 7 Distance. In contrast, Scrum-halves did not present any differences in high-intensity speed parameters with Centres. Fly-halves showed statistically lower values in Maximum Velocity, Velocity Band 7 Distance, and Effort Count than Centres and had lower values compared to the Back-three players in Velocity Band 7 Distance and Effort Count. However, the Centres did not show any significant differences compared to Back-three for the high-intensity speed parameters.

### 3.2. Principal Component Analysis

The PCA revealed different models that explained 84,65% of the total variance (Table 4), obtaining four categories or components of speed performance parameters (Table 5).

- Factor 1 was named “High Intensity” speed performance parameters positively correlated with Velocity Band 4-5-6-7 Distance and Velocity Band 4-5-6-7 Effort Count parameters, corresponding to speeds between 15 and 30 km/h.
- Factor 2 was identified as “Low Intensity” speed performance parameters composed of Velocity Band 1-2-3 Distance and Velocity Band 2-3 Effort Count, corresponding to speed between 0 and 15 km/h, Total distance, and Total PlayerLoad. It is also positively correlated to PlayerLoad per Minute and Meters per Minute.
- Factor 3 was recognized as the “Max Intensity” speed performance parameter, strongly and positively correlated with Velocity Band 8 Distance and Effort Count, corresponding to speeds greater than 30 km/h. Note that the top speed observed in the dataset was 34.09 km/h. It is also positively correlated with Velocity Band 7 Distance and Effort Count, corresponding to a speed between 25.2 and 30 km/h.
- Factor 4 was labeled as “Time Related” speed performance parameters, collecting variables such as Played Time, Meters per Minute, and PlayerLoad per Minute.

### 3.3. Players’ Profile

The two-stage cluster analysis performed with the twenty-two PC entries from the PCA resulted in three clusters, classifying the entire sample. The first one, “Low-Demands”, grouped 35.5% of the sample, the second cluster, “Mid-Demands”, grouped 38.4% of the sample, and the third cluster, “High-Demands”, grouped 26.1% of the sample. The most important predictors, with importance values greater than 0.70 in the clustering model were Velocity Band 7 Distance (1) and Effort Count (0.98), Velocity Band 6 Effort Count (0.89) and Distance (0.87), Total Distance (0.84), Velocity Band 5 Effort Count (0.77) and Distance (0.74), and Maximum Velocity (0.71). Table 6 presents the descriptive statistics (mean and predictor importance) of the most important variables (importance > 0.70) that determined cluster classification [31].

**Figure 1:**
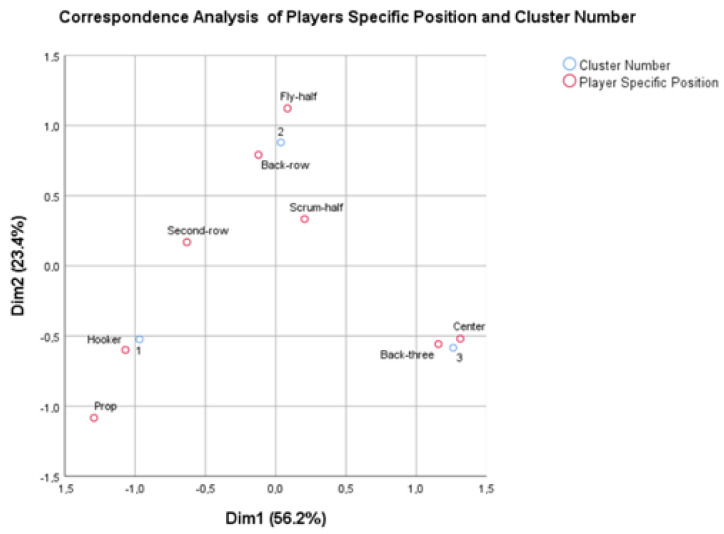
Symmetric two-dimensional correspondence analysis (CA) map of player’s specific position according to the previous clusters.

The correspondence analysis showed a graphical representation of the specific positions of the player according to the resulting low-, mid-, and high-physical demand clusters (Figure 1). The sum of the proportion of variance explained by the vertical and horizontal dimensions, the CA, explained 79.6% of the variance in the data.

## 4. Discussion

Despite the existing literature on the physical demands of elite rugby union, most studies have focused on Tier 1 national teams or professional clubs, with limited attention to Tier 2-level international competition. Additionally, the current evidence rarely addresses the need for individualized training programs based on positional demands in these contexts. Understanding these demands is critical for tailoring training loads and optimizing performance.

Thus, to the authors’ knowledge, this is the first study to investigate the international level of Tier 2 rugby union teams. The purposes of this research were to recognize differences in specific player positions in speed performance parameters, to understand the principal components of speed performance parameters, and to create a model of position profiles through the PCs of the speed performance parameters. Through PCA, a total of 22 PCs of the whole dataset of speed performance parameters were extracted, revealing four different models, explaining 84,65% of the total variance: Max Intensity, High Intensity, Low Intensity, and Time Related speed performance parameters. The entire sample was then classified using cluster analysis. This grouped the different players into three groups: high, medium, and low speed demands, as shown in Figure 1.

In the present study, PCA’s factor 1, labeled “High Intensity”, explained 49.61% of the variance from the dataset of speed parameters, corresponding to the running distance and effort bouts between 15 and 30 km/h. This revealed the importance of high-intensity running in rugby performance, supported by the existing scientific literature focusing on the high-intensity parameters [2,8,9,10].

Few investigations have used the analysis of PCs in rugby union [13,14]. A 2020 study interested in university rugby union level used PCA to identify the movement demands in university-level rugby union by analyzing all metrics extracted by GPS technology [32]. According to our results, we found a High-Intensity Running (HIR) model positively correlated with sprinting, total high-speed distance (sum of HIR 18.0–21.6 km/h, and sprinting > 21.6 km/h) corresponding to velocity bands 5 and 6 in our study. The HIR model also correlated with High-Intensity Acceleration (HIA) and relatively high-intensity efforts.

In our investigation, the “High Intensity” factor was more important than the third factor called “Max Intensity”, explaining just 8.46% of the variance, highly correlated with velocity band 8 distance and effort count (>30 km/h). This can be explained by the low data volume found in that specific velocity band: only 52 GPS files from n = 230 performed these high-intensity efforts, totaling 101 efforts with a mean of 0.46 ± 0.97 efforts produced per file.

In this research, cluster analysis was used to group different specific player positions according to the PCs extracted from the speed performance parameters. Little research has used these statistical models applied to rugby union. The biomechanical parameters of the place kick of professional rugby players and collisions of both amateur and professional players in all age categories have been studied with this technique [17,18]. Also, this technique has been performed over a battery of conditioning-specific movement tasks (conditioning movements, sprinting, jumping, and endurance test) to group rugby players into different profiles of competencies [16] or to expose the differences between positions, showing 10 specific positions (5 forwards and 5 backs) extracted from movements and activities of players in international rugby union matches [4].

Two clusters of youth rugby union players were found analyzing total distance covered, distance covered at different speed bands, heart rate, and number of impacts [33]. Contrary to the present research, that study showed that the most important predictors were heart rate-related variables and distance covered at low intensity (under 12.9 km/h). The difference in sample age could explain this. However, it was observed that one of the clusters tended to show higher values than the other in higher-speed zones.

Differences between forwards and backs have been described by the existing literature focusing on movement demands and work–rest ratios [1,32], impacts [34,35], age group [36], or influenced by fatigue [37]. In this study, the back-specific positions were more homogeneous among them than the forward-specific positions. The most notorious differences found in this research between forwards and backs were in the distance covered and the number of efforts made at high intensity, from band 5 to 8, which corresponds to 18 to 39.6 km/h. The literature confirms that backs have a much higher requirement for high-speed running demands in many variables than forwards, considering a high-intensity running threshold of 5 m/s or 18 km/h [22].

Within the forward group, the results showed that the Props and Hookers had similar speed demands but differed from Second-row players, who have comparable demands with Back-row players. On the other hand, Fly-halves had similar speed demands as Scrum-halves, and Centres had similar demands to the Back-three players (Figure 1). In 2015, professional rugby players showed differences between tight forwards (Prop, Hooker, Second rows) and loose forwards (Open-side Flanker, Blind-side Flanker, No 8), while half-backs (Scrum-half, Outside-half) showed different distances covered in sprinting and high-intensity running compared with inside-backs (inside-centre, outside-centre) and outside-backs (wingers, fullback) [19].

The findings presented in this research offer practical applications for athlete health monitoring and injury prevention. By classifying players into high-, mid-, and low-demand clusters based on their specific speed-related profiles, practitioners can tailor workload management strategies to the physiological demands of each positional group. The identification of high-speed and high-effort running profiles—particularly in backs—suggests a greater need for structured load progression, individualized recovery protocols, and targeted conditioning programs to mitigate injury risk. Conversely, the comparatively lower-intensity profiles observed in certain forward positions may warrant distinct emphasis on contact-skill-related load monitoring and strength-based conditioning. Overall, the integration of multivariate speed metrics through PCA and clustering supports a more nuanced and evidence-based approach to injury prevention and performance optimization in Tier 2 rugby union contexts and provides a foundation for designing position-specific training periodization and injury prevention strategies.

The classification of players into Low-, Mid-, and High-Demands clusters may also help coaches and performance staff to adjust recovery strategies depending on the specific demands of each player group. For example, players in the High-Demands cluster, usually backs, who perform more high-speed and high-effort running actions, may benefit from individual recovery plans such as cold-water immersion or active recovery, which have been shown to accelerate neuromuscular recovery within 48 h after maximal efforts [38,39]. In contrast, players in the Low-Demands cluster, often forwards, may require a focus on collision-related loading and strength-based conditioning. Monitoring collision loads through GPS data is important, as high acute–chronic workload ratios have been associated with a higher risk of injury [40]. Also, combining GPS training load with players’ daily wellness ratings has been used to adjust training in rugby and support individual recovery decisions [41]. Therefore, using demand-based clusters can help coaches to better plan workload and recovery, improving performance and reducing the risk of injury in Tier 2 rugby union players.

This study was conducted on a sample of international matches of a national team ranked as a Tier 2 nation. Gabbett described [42] that physical performances tend to rise with higher levels of competition in rugby league, especially in sprints. Furthermore, significantly higher values were found for repeated high-intensity locomotion efforts (RHILE) for each position studied at the international level compared to club games [21]. This might support the idea of an existing relationship between higher physical demands and higher competition levels. However, further investigations might be conducted to clarify this possibility at the given international level.

This study presents additional limitations, such as analyzing a single Tier 2 national team, which may restrict the generalization of the results. Furthermore, the team studied competed against both Tier 2 and Tier 3 nations, which could have influenced the physical performance parameters. Nevertheless, the Spanish team regularly participates at the Tier 2 international level and is composed of professional players, which reinforces the relevance of the findings for this competitive context. Although caution is necessary when extrapolating the outcomes to other Tier 2 teams, the positional trends observed, such as differences in speed performance parameters and greater high-intensity running demands in backs, are aligned with previous findings from higher-level competitions. Future investigations, including a broader sample of Tier 2 national teams, are recommended to improve external validity and verify whether the current findings are representative of the wider Tier 2 rugby union environment.

## 5. Conclusions

Despite the paucity of evidence and research on international Tier 2 rugby union, the specific knowledge extracted in this study may support a more individualized prescription of training programs. This could be applied by following the inter-positional trend of different groups of players clustered by the specific speed demands. This study’s results also highlight the potential of using speed-based demand clusters to inform individualized injury prevention strategies and optimize recovery protocols tailored to the specific physical profiles of Tier 2 rugby union players.

Therefore, results from the present investigation can generate a better understanding for coaches and professional staff of the external loads that different specific positions may have and what variables express a significant proportion of the dataset variance for this objective. Future research about this international rugby union level should be conducted to contextualize and compare these findings.

## Figures and Tables

**Table 1:** Descriptive data as mean (M) and standard deviation (SD) of the subject’s anthropometry.

	Height (Meters)		Body Mass (Kilograms)	
	M	SD	M	SD
Prop (n = 6)	1.86	0.06	116.50	10.11
Hooker (n = 5)	1.84	0.08	105.20	1.64
Second-row (n = 7)	1.92	0.07	108.43	6.50
Back-row (n = 9)	1.89	0.05	104.11	3.22
Scrum-half (n = 5)	1.74	0.03	73.80	4.82
Fly-half (n = 4)	1.80	0.07	83.25	5.38
Centre (n = 6)	1.85	0.06	90.83	6.31
Back-three (n = 13)	1.82	0.06	88.69	8.22

Note: “n”: number of players included in the study for each specific player’s position.

**Table 2:** Variables used in the study.

Variable	Definition
Played Time	Total duration of the match (minutes)
Total Distance	Distance covered during the match (meters)
Meters Per Minute	Total distance covered divided by minutes in the selected split (meters/minute)
Total PlayerLoad	The sum of the accelerations across all axes of the internal tri-axial accelerometer during movement divided by a scaling factor (arbitrary units)
PlayerLoad Per Minute	The sum of the accelerations across all axes divided by a scale factor (100) and divided by minutes in the selected split (arbitrary units)
Velocity Band Distance	Total distance (meters) covered within the specific velocity band per period
Velocity Band Effort Count	Total amount of times the specific velocity band was reached per period
Maximum Velocity	Maximum velocity recorded (km/h)
Sprint Distance	Percentage of the distance accumulated while running at high speed/sprinting > 21.6 km/h (percentage)

Notes: general speed parameters (Played Time, Total Distance, Meters per Minute, Total PlayerLoad, PlayerLoad per Minute), low-intensity speed parameters (Velocity Band 1 to 4 Distance and Effort Count), high-intensity speed parameters (Velocity Band 5 to 8 Distance and Effort Count, Maximum Velocity, and Sprint Distance).

**Table 3:** Descriptive data for the speed performance parameters, separated by specific playing position. Data are presented as n, mean, and standard deviation.

Position	Prop (A)		Hooker (B)		Second-Row (C)		Back-Row (D)		Scrum-Half (E)		Fly-Half (F)		Centre (G)		Back-Three (H)	
	(n = 24)		(n = 12)		(n = 37)		(n = 41)		(n = 20)		(n = 17)		(n = 30)		(n = 49)	
	M	SD	M	SD	M	SD	M	SD	M	SD	M	SD	M	SD	M	SD
Played Time	69.4	10.7	68.4	9.30	83.1	12.6 a **, b *	88.5	12.7 a ***, b **	83.7	6.73 a **, b **	80.0	13.5	88.6	10.9 a ***, b **	86.3	11.3 a ***, b **
Total Distance	4013	553	4458	731	4747	1216 a *	5877	1108 a ***, b **, c **	5163	1385 a *	5924	1365 a ***, b *	5751	1224 a ***, b *, c *	6189	1275 a ***, b ***, c ***
Meters Per Minute	58.8	8.86	66.5	10.3	58.7	9.12 b *	66.3	7.63 a *, c ***	62.1	17.1	75.0	15.5 a **, c ***, d **, e *	65.6	12.8 a *, c **, f *	71.6	1.3 a ***, c ***, d **
Total PlayerLoad	439	53.9	508	77.8	511	118 a *	625	127 a ***, c **	507	139	550	108 a **	541	129 a **	552	112 a ***
PlayerLoad Per Minute	6.43	0.906	7.47	1.02 a *	6.34	0.881 b *	7.05	1.01 c*	6.11	1.78	6.96	1.21	6.27	0.887 b **, d *	6.40	1.08 b *
Velocity Band 1 Distance	2349	413	2376	302	2666	828	3241	627 a ***, b ***, c *	2415	636 d ***	2936	715	3087	695 a **, b *, e *	3344	786 a ***, b ***, c **, e ***
Velocity Band 2 Distance	761	131	789	298	839	238	975	229 a **	760	244 d ***	924	199	857	229	876	218
Velocity Band 2 Effort Count	98.8	17.4	115	33.4	126	32.3 a **	147	29.9 a ***, b *	128	45.6	135	35.8 a *	128	33.6 a **	133	29.1 a ***
Velocity Band 3 Distance	688	181	952	327	799	266	1001	338 a **	981	354	1090	351 a **	818	240	919	270 a **
Velocity Band 3 Effort Count	53.4	12.3	74.1	20.4 a *	68.0	20.2 a *	86.8	22.0 a ***, c *	96.0	32.0 a **, b **	91.1	24.4 a ***, c *	80.3	23.0 a ***	87.8	21.7 a ***, c **
Velocity Band 4 Distance	139	46.0	238	84.0 a *	262	113 a ***	352	154 a ***, b *	517	173 a ***, b ***, c ***, d *	523	171 a ***, b **, c ***	409	132 a ***, b **, c ***	402	124 a ***, b **, c ***
Velocity Band 4 Effort Count	13.1	4.58	19.8	6.45	23.3	9.54 a ***	30.3	12.8 a ***	43.0	17.0 a ***, b **, c ***	40.5	12.2 a ***, b **, c ***	37.4	12.1 a ***, b ***, c ***	35.3	10.8 a ***, b ***, c ***
Velocity Band 5 Distance	58.2	25.2	79.3	40.	122	79.6 a *	209	106 a ***, b **, c **	292	129 a ***, b **, c ***	296	132 a ***, b **, c ***	302	139 a ***, b ***, c ***	307	135 a ***, b ***, c ***, d *
Velocity Band 5 Effort Count	4.83	1.93	6.17	3.07	9.68	6.46 a *	16.1	7.70 a ***, b **, c **	21.9	9.90 a ***, b **, c ***	23.0	10.6 a ***, b **, c ***	23.1	10.9 a ***, b ***, c ***	24.1	11.7 a ***, b ***, c ***, h *
Velocity Band 6 Distance	13.6	11.4	18.9	11.8	40.7	36.5 a *	75.2	56.5 a ***, b *	119	75.0 a ***, b ***, c ***	118	69.2 a ***, b **, c ***	159	71.4 a ***, b ***, c ***, d ***	178	80.3 a ***, b ***, c ***, d ***
Velocity Band 6 Effort Count	1.21	1.02	1.25	1.06	2.92	2.53	5.44	3.93 a ***, b **	8.50	5.61 a ***, b ***, c ***	7.71	5.01 a ***, b **, c **	10.7	5.86 a ***, b ***, c ***, d ***	12.5	6.95 a ***, b ***, c ***, d ***
Velocity Band 7 Distance	4.00	9.24	3.54	6.76	16.1	29.4	18.6	28.2	64.2	61.4 a ***, b **, c **, d *	35.0	32.0 a ***, b *	89.4	51.3 a ***, b ***, c ***, d ***, f ***	117	58.4 a ***, b ***, c ***, d ***, e *, f ***
Velocity Band 7 Effort Count	0.292	0.751	0.250	0.622	1.05	1.82	1.17	2.06	4.30	4.17 a ***, b **, c *, d **	2.00	1.50 a ***, b *	5.83	3.42 a ***, b ***, c ***, d ***, f *	7.67	4.49 a ***, b ***, c ***, d ***, f ***
Velocity Band 8 Distance	0.00	0.00	0.273	0.944	1.83	5.50	4.35	13.3	9.08	16.5 a *	2.19	5.07	10.2	12.7 a ***, c **, d **	10.4	13.2 a ***, c **, d **
Velocity Band 8 Effort Count	0.00	0.00	0.083	0.289	0.0811	0.277	0.293	0.814	0.789	1.36	0.176	0.393	0.889	1.15 a **, c **, d *	1.05	1.30 a ***, c ***, d **
Maximum Velocity	22.4	1.77	23.2	1.48	23.5	2.07 b *	25.4	1.82 a *, c ***	26.6	1.68	27.2	1.78 a **, c ***, d **, e *	29.1	2.63 a *, c **, f *	30.3	2.29 a ***, c ***, d **
Sprint Distance	0.361	0.316	0.452	0.301	0.864	0.756	1.28	0.899 a ***	2.21	1.04 a ***, b ***, c ***	1.96	1.02 a ***, b **, c **, d *	2.69	1.10 a ***, b ***, c ***, d ***	2.81	1.16 a ***, b ***, c ***, d ***

Notes: Statistical differences are presented as (a) for Props, (b) for Hookers, (c) for Second-row players, (d) for Back-rows, (e) for Scrum-half, (f) for Fly-half, (g) for Centres, (h) for Back-three and are shown as * = *p* < 0.05; ** = *p* < 0.01; *** = *p* < 0.001. (The explanation of letters is not case sensitive).

**Table 4:** Eigenvalues for components and variance are explained by the outcome.

Factors	Initial Eigenvalues			Extraction Sums of Squared Loadings			Rotation Sums of Squared Loadings		
	Total	% of Variance	% Cumulative	Total	% of Variance	% Cumulative	Total	% of Variance	% Cumulative
High intensity	10.91	49.61	49.61	10.91	49.61	49.61	7.42	33.71	33.71
Low intensity	4.49	20.40	70.01	4.49	20.40	70.01	6.86	31.18	64.89
Max Intensity	1.86	8.46	78.47	1.86	8.46	78.47	2.86	13.02	77.91
Time Related	1.36	6.18	84.65	1.36	6.18	84.65	1.48	6.74	84.65

**Table 5:** Rotated component for the speed performance parameters.

	High Intensity	Low Intensity	Max Intensity	Time Related
Played Time				−0.721
Total Distance		0.854		
Total PlayerLoad		0.921		
PlayerLoad Per Minute		0.617		0.693
Meters Per Minute		0.618		0.647
Maximum Velocity	0.714			
Velocity Band 1 Distance		0.744		
Velocity Band 2 Distance		0.878		
Velocity Band 3 Distance		0.834		
Velocity Band 4 Distance	0.721			
Velocity Band 5 Distance	0.902			
Velocity Band 6 Distance	0.921			
Velocity Band 7 Distance	0.664		0.626	
Velocity Band 8 Distance			0.927	
Velocity Band 2 Effort Count		0.923		
Velocity Band 3 Effort Count		0.806		
Velocity Band 4 Effort Count	0.761			
Velocity Band 5 Effort Count	0.917			
Velocity Band 6 Effort Count	0.903			
Velocity Band 7 Effort Count	0.642		0.631	
Velocity Band 8 Effort Count			0.906	
Sprint Distance	0.919			

**Table 6:** Predictor importance and descriptive data (mean, SD) for each cluster.

Predictor	Predictor Importance	Cluster I Low-Demands		Cluster II Med-Demands		Cluster III High-Demands	
Velocity Band 7 Distance	1	10.72	20.41	18.8	20.15	108.18	42.16
Velocity Band 7 Effort Count	0.98	0.68	1.39	1.12	1.28	6.75	2.64
Velocity Band 6 Effort Count	0.89	1.88	1.95	6.09	3.63	14.94	5.51
Velocity Band 6 Distance	0.87	25.69	26.36	89.34	56.67	202.12	68.02
Total Distance	0.84	3958.02	852.97	6027.19	821.02	6403.32	821.79
Velocity Band 5 Effort Count	0.77	6.65	4.09	18.78	8.22	29.49	9.45
Velocity Band 5 Distance	0.74	83.22	51.57	246.87	110.97	371.89	119.64
Maximum Velocity	0.71	23.27	1.97	26.02	2.46	29.56	1.89

Notes: descriptive data of distance are expressed in meters, effort count in number of bouts, and Maximum Velocity in kilometers/hour.

## Data Availability

The data presented in this study are available on request from the corresponding author.

## Abbreviations

The following abbreviations are used in this manuscript:

GPS	Global Positioning System
RHIE	Repeated High-Intensity Efforts
BiP	Ball in Play
WCS	Worst-Case Scenario
SSG	Small-Sided Game
PCA	Principal Component Analysis
PC	Principal Component
REC	European Rugby Championship
AU	Arbitrary Units
KMO	Kaiser–Meyer–Olkin
CA	Correspondence Analysis
IBM	International Business Machines
SPSS	Statistical Package for the Social Sciences
UAX	Universidad Alfonso X el Sabio
FER	Federación Española de Rugby
